# Does governance play a role in the distribution of invasive alien species?

**DOI:** 10.1002/ece3.3744

**Published:** 2018-01-17

**Authors:** Thomas Evans, Philine zu Ermgassen, Tatsuya Amano, Kelvin S.‐H. Peh

**Affiliations:** ^1^ Conservation Science Group Department of Zoology University of Cambridge Cambridge UK; ^2^ Aquatic Ecology Group Department of Zoology University of Cambridge Cambridge UK; ^3^ Centre for the Study of Existential Risk University of Cambridge Cambridge UK; ^4^ Biological Sciences University of Southampton Southampton UK; ^5^ Present address: School of Geosciences University of Edinburgh Edinburgh UK

**Keywords:** corruption, DAISIE, environmental governance, human movement, propagule pressure, tourism, trade, travel, worldwide governance indicators

## Abstract

Invasive alien species (IAS) constitute a major threat to global biological diversity. In order to control their spread, a detailed understanding of the factors influencing their distribution is essential. Although international trade is regarded as a major force structuring spatial patterns of IAS, the role of other social factors remains unclear. Despite studies highlighting the importance of strong governance in slowing drivers of biodiversity loss such as logging, deforestation, and agricultural intensification, no study has yet analyzed its contribution to the issue of IAS. Using estimates of governance quality and comprehensive spatiotemporal IAS data, we performed multiple linear regressions to investigate the effect of governance quality upon the distribution of species listed under “100 of the worst” IAS in 38 Eurasian countries as defined by DASIE. Our model suggested that for countries with higher GDP, stronger governance was associated with a greater number of the worst IAS; in contrast, for the lowest GDP countries under analysis, stronger governance was associated with fewer of these IAS. We elucidate how the quality of governance within a country has implications for trade, tourism, transport, legislation, and economic development, all of which influence the spread of IAS. While our findings support the common assumption that strengthening governance benefits conservation interventions in countries of smaller economy, we find that this effect is not universal. Stronger governance alone cannot adequately address the problem of IAS, and targeted action is required in relatively high‐GDP countries in order to stem the influx of IAS associated with high volumes of trade.

## 1 INTRODUCTION

Effective prevention and control of invasive alien species (IAS) require a thorough understanding of the determinants of invasion. The socioeconomic context of biological invasions is thought to be well understood, with previous studies highlighting the role of international trade (Westphal, Browne, MacKinnon, & Noble, 2008) and globalization (Amano, Coverdale, & Peh, 2016; Meyerson & Mooney, 2007). One socioeconomic factor of biological invasions, however, has not yet been addressed in this context. Despite an emerging literature examining the role of governance in issues such as biodiversity loss (Smith, Muir, Walpole, Balmford, & Leader‐Williams, 2003), illegal logging (Smith, Obidzinski, Subarudi, & Suramenggala, 2003), agricultural intensification (Ceddia, Bardsley, Gomez‐y‐Paloma, & Sedlacek, 2014), and deforestation (Umemiya, Rametsteiner, & Kraxner, 2010), there is a paucity of studies considering its role during the process of biological invasion (Lotz & Allen, 2013).

Governance is defined as, “the traditions and institutions by which authority in a country is exercised” (Kaufmann, Kraay, & Mastruzzi, 2011). Biodiversity conservation is intimately related to multiple aspects of governance. For example, corruption has been correlated with changes in forest cover, numbers of African elephants, and numbers of black rhinoceroses, illustrating that strong governance is essential to slowing the rate of biodiversity loss (Smith, Muir, et al., 2003). Similarly, illegal logging in Indonesia increased during political transitions, when governments are weak and have underdeveloped institutions, and thus more vulnerable to corruption (Smith, Obidzinski, et al., 2003). In support of this, worsening corruption correlated with poorer environmental performance across 66 tropical developing countries worldwide (Peh & Drori, 2010), and increases in deforestation rates have been found to associate with decreases in the quality of governance (Umemiya et al., 2010).

Besides control of corruption, there are other aspects of governance, such as Political Stability, Voice and Accountability, Government Effectiveness, Regulatory Quality, and Rule of Law (Kaufmann et al., 2011). There has been little focus to date on what role these aspects of governance play in the distribution of IAS. Only one previous study has briefly explored the impact of governance upon invasive species (Lotz & Allen, 2013), finding some evidence of a relationship between Political Stability (as defined and estimated by Kaufmann, Kraay, & Mastruzzi, 2009) and the prevalence of invasive birds and mammals in 100 countries worldwide. But in other environmental studies, political instability, in combination corruption, has been found to reduce the stringency of environmental regulations (Fredriksson & Svensson, 2003). Likewise, stronger democracy—through its protection of free speech and its capacity to hold leaders accountable—has been shown to reduce aquatic pollution, deforestation, and land degradation (Li & Reuveny, 2006), whilst increasing protected land area (Midlarsky, 1998).

Whether stronger governance plays a beneficial role concerning IAS remains unknown. In this study, we used cross‐country estimates on the quality of governance in conjunction with comprehensive Eurasian IAS data to explore the role of governance in structuring the distribution of the 100 “worst”’ (i.e., most severe) IAS for 38 Eurasian countries, as defined by DASIE. Increased trade and economic development have been shown to correlate with more heavily invaded countries (Pyšek et al., 2010; Westphal et al., 2008). We hypothesized that stronger governance—through its ability to foster a society which enforces environmental laws and can effectively monitor and regulate IAS—acts to mitigate the effect of trade on introducing damaging IAS between economically developed countries. In contrast, economically equivalent countries with comparatively poorer governance would suffer relatively more invasions.

## 2 METHODS

### 2.1 Governance data

Indicators for six dimensions of governance were taken from the Worldwide Governance Indicators Project (WGI). These six aggregate indicators are weighted averages of data collated from hundreds of individual variables measuring governance worldwide (Kaufmann et al., 2011), making them a comprehensive measure of governance. The six indicators are (adapted from Kaufmann et al., 2011) as follows:

1. Voice and Accountability—freedom of expression and the extent to which citizens participate in government matters.
2. Political Stability and Absence of Violence—the likelihood of political instability and terrorism.
3. Government Effectiveness—the quality of policy formulation and implementation.
4. Regulatory Quality—the quality of private sector regulation.
5. Rule of Law—the extent to which people trust and abide by the rules of society, including the quality of contract enforcement, property rights, the police, and the courts.
6. Control of Corruption—the extent to which public power is exercised for private gain.

**Figure 1:**
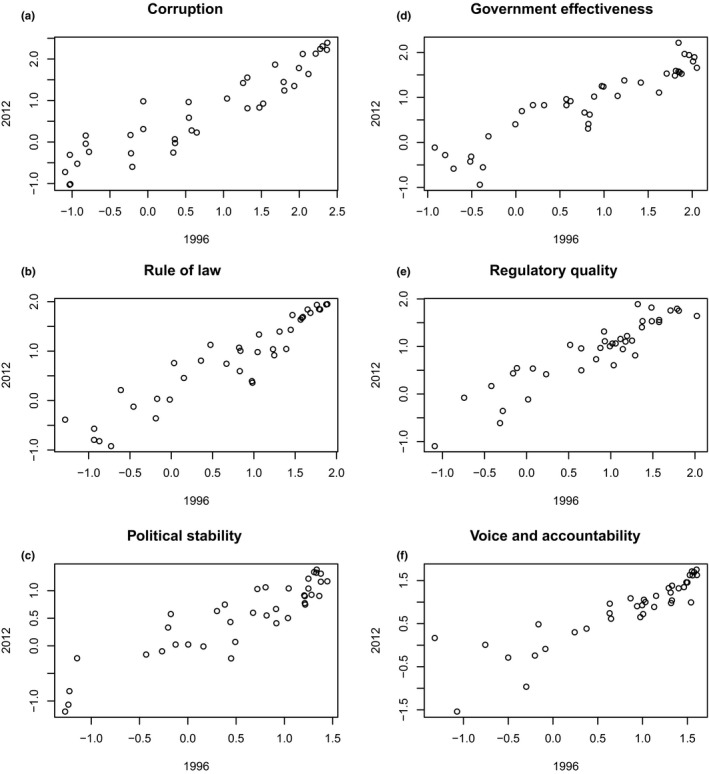
Governance estimates from 1996 to 2012 in six aggregate indicators (a–f) were examined using Spearman's rank correlation tests (*r*_s_). In all six indicators, estimates from 1996 were correlated with estimates from 2012. (a) Corruption: *r*_s_ = .931. (b) Rule of Law: *r*_s_ = .947. (c) Political Stability: *r*_s_ = .875. (d) Government Effectiveness: *r*_s_ = .940. (e) Regulatory Quality: *r*_s_ = .908. (f) Voice and Accountability: *r*_s_ = .932. In all correlations, *n* = 38, *p* < .001

The concept of “invasion debt”—in which current patterns of IAS richness are better described by historical rather than modern socioeconomic data (Essl et al., 2011)—suggests that contemporary indicators are unsuitable for this analysis because they fail to reflect governance at the time of an introduction. We therefore first explored whether the relative rank of countries’ governance changed over time. While WGI only began in 1996, changes in WGI estimates from 2000 to 2009 are small worldwide (Kaufmann et al., 2011), and from 1998 to 2008, just 29% of countries under our analysis showed significant changes in a *single* aggregate indicator (Kaufmann et al., 2009), indicating that governance estimates are surprisingly static over short timescales. As evidence, WGI estimates from 1996 were compared to 2012 estimates using Spearman's rank correlation tests. For all six WGI indicators, 1996 estimates strongly correlated with those in 2012 (Figure 1), confirming that governance data are relatively stable over time.

To support this assumption across longer timescales and throughout major changes in the Eurasian political landscape, we confirmed this result using an older, alternative governance index: the International Country Risk Guide. Data for the ICRG's Political Risk Ratings (PRS Group) are available since 1984. The ICRG is the earliest, publicly available governance indicator, representing an upper limit on the historical availability of governance estimates (Kaufmann et al., 2009). For each country, earliest available annual mean ratings were compared to mean scores from 2012 using Spearman's correlations; former nations were compared against modern counterparts (e.g., Slovakia and the Czech Republic were matched against Czechoslovakia). Scores from 1984 to 1986 were significantly correlated with ratings from 2013 (*r*_s_ = .697, *n* = 31, *p* < .001), reinforcing the description of governance estimates as time‐invariant. This assumption of time invariance in governance has similarly been justified in a previous study of governance and biodiversity (Ceddia et al., 2014), further increasing our confidence in this instance.

Numerous studies within the governance‐biodiversity literature have analyzed individual WGI indicators, and it is clear that there are benefits of having insight into how individual aspects of governance interrelate with biodiversity (Ceddia et al., 2014; Eklund, Arponen, Visconti, & Cabeza, 2011; Lotz & Allen, 2013). As such, we analyzed individual WGI indicators separately, as well as an overall score for governance. As in a previous study (Umemiya et al., 2010), an overall governance score (from 0 [low] to 30 [high]) was calculated by summing mean scores during the period 1996–2012 for all six indicators, and setting these values relative to zero. Lower scores correspond to relatively weaker governance; higher scores correspond to relative stronger governance.

### 2.2 Alien species data

Invasive alien species introduced into Eurasia prior to 1952 were excluded from the analysis. The earliest international treaty targeting IAS in Eurasia was the 1951 International Plant Protection Convention, in force since 1952 (Genovesi & Shine, 2003). Although governance constitutes more than legislation, the existence of legislation and compliance to it encompasses numerous aspects of governance including Rule of Law, Government Effectiveness, Control of Corruption, and Regulatory Quality. The existence of legislation is therefore a good indicator that *intentional* governance mechanisms were in existence in relation to IAS. The international treaty is a good cross‐country indicator of the intention to govern as it establishes that in 1952 Eurasian nations were aware of IAS and had policy in place to target IAS. Therefore, any new introductions post‐1952 have the potential to be influenced by governance. Introductions prior to 1952 were therefore excluded from the study, since these would have occurred outside a timeframe in which it would be guaranteed that governance could have had any intentional effect.

We are aware that including all species that have been recorded since 1952 undoubtedly means that some species recorded arrived before the convention was in place, due to the lag between arriving and recording of invasive species (Jeschke & Strayer, 2005; Kowarik, 1995). Nevertheless, as the lag time is both highly variable and unpredictable (Jeschke & Strayer, 2005; Kowarik, 1995), we felt this was the best strategy for capturing all the species introduced after the convention came into force.

Data on IAS were collated from the DAISIE European Invasive Alien Species Gateway (http://www.europe-aliens.org). Although the most comprehensive IAS database for Eurasia (including non‐European countries such as Russia, Israel, and Turkey), country‐specific information on the timing of introductions for most species is either poor or absent. It was therefore not possible to analyze governance in relation to *total* IAS richness per country. However, DAISIE provides extensive information, including known arrival dates for each country, for a subset of IAS identified by DAISIE as being “100 of the worst” IAS in Eurasia in terms of their severely negative impact on biodiversity, economy, and public health. This dataset (hereinafter “DAISIE 100”) informs about the distribution of the 100 worst IAS in Eurasia.

The DAISIE 100 represents species from a broad suite of taxa and habitats, including 18 terrestrial plants, 16 terrestrial invertebrates, 15 terrestrial vertebrates, 16 inland water species, three terrestrial fungi, and 32 species from coastal waters, thereby representing all main taxonomic groups and all environments (Vilà et al., 2009). DAISIE 100 species pose significantly severe threats to biodiversity: 71% are recorded to have reduced native biodiversity or altered the invaded community, and 19% have threatened endangered species (Vilà et al., 2009). Therefore, despite the absence of country‐specific information on their impacts, the DAISIE 100 is a suitable proxy for the invasive richness in a country, especially since the number of 100 worst species per country correlates with total IAS richness (*r*_s_ = .730, *p* < .001, *n* = 38). As not all countries have coastal areas, marine species are a potential source of bias and were therefore excluded from the analysis, leaving 68 species. For each country, a DAISIE 100 score was calculated by counting the number of species on the “100 worst” list introduced from 1952 onwards, excluding marine species. Higher scores indicate countries have more invasions.

### 2.3 Explanatory variables

Thirteen explanatory variables, including governance, were considered to account for factors known to influence the distribution of IAS. (Hayes & Barry, 2008), and thus, their inclusion in the model was warranted. One explanatory variable was governance, both as an overall governance score and as six separate indicators.

Of the 12 remaining explanatory variables, three captured climatic factors, since climate matching between native and introduced ranges is important in determining the distribution of invasive mammals (Forsyth, Duncan, Bomford, & Moore, 2004); reptiles and amphibians (Bomford et al. 2009); fish (Bomford et al. 2010); plants, insects, shellfish, and finfish. A further four variables captured economic factors, because economic activity is well studied as a driver of the proliferation of exotic species (Taylor & Irwin, 2004). The final five explanatory variables captured natural and human geographies which also shape the dynamics of IAS.

These 12 explanatory variables, and the justification for their inclusion, are listed here:

1. Area (km^2^, Central Intelligence Agency 2014) is a strong predictor of IAS richness per country (McGeoch et al., 2010).
2. Continentality (difference in mean January and June temperature, °C; Mitchell, Carter, Jones, Hulme, & New, 2004) was expected to correlate negatively with the worst IAS: lower annual variability in temperature might give IAS a stronger likelihood of finding the climate favorable for establishment.
3. Mean annual precipitation (mm; Mitchell et al., 2004) and
4. Mean annual temperature (°C; Mitchell et al., 2004) has been previously found to determine IAS richness (Lambdon et al., 2008).
5. Insularity (island = 1; mainland = 0) affects the distribution of IAS, since islands are typically more heavily invaded than mainland (Simberloff, 1995).
6. Human Population (The World Bank, 2013) and
7. Human Population Density (people/km^2^ of land area, The World Bank, 2013) are known to account for IAS richness across countries (McKinney, 2006; Pyšek et al., 2010) and were therefore included in our model.
8. Road Density (km road/100 km^2^ of land area, The World Bank, 2013) facilitates the dispersal of IAS (Hulme 2009. For example, the extent of terrestrial transport networks accounted for the density of alien plants in European and North African countries (Vilà & Pujadas, 2001), justifying its inclusion in the analysis.
9. GDP (US$, The World Bank, 2013) positively associates with the number of invasive plants, birds, fish, and mammals in Europe (Hulme, 2007).
10. GDP per capita (US, The World Bank, 2013) has accounted for the global spread of invasive birds, and mammals (Lotz & Allen, 2013).
11. Merchandise Imports (US$, The World Bank, 2013) has been previously used in IAS studies to capture the extent of a country's participation in international trade (Westphal et al., 2008). Trade has been shown to account for global patterns in IAS (Westphal et al., 2008).
12. The KOF Index of Globalisation reflects the economic, political, and social dimensions of globalization for a country (Dreher, 2006). Globalization is argued to be accelerating the rate of biological invasion worldwide and thus merits inclusion in our analysis (Meyerson & Mooney, 2007).

Direct measures of ecosystem disturbance, which are known to facilitate the establishment of IAS (Lozon & MacIsaac, 1997), have not been included. Although percentage agricultural land has been previously used as a measure of habitat disturbance (Lotz & Allen, 2013), only a small proportion of species used to calculate DAISIE 100 scores were found in agricultural habitats (29%), making it unsuitable to use here. Furthermore, GDP has been previously used as a proxy for disturbance (Westphal et al., 2008), suggesting it will be accounted for in our models indirectly.

### 2.4 Statistical analysis

Before constructing the global model, explanatory variables in the multiple linear regressions were examined for collinearity with Pearson's tests (Table S1). The less‐informative parameter of strongly correlated variables (*r* > .7 or < −.7) was eliminated. GDP correlated with Merchandise Imports (*r* = .951, *p* < .001) and Human Population (*r* = .925, *p* < .001); as these were also correlated with each other (*r* = .846, *p* < .001), both were excluded from the analysis. As Merchandise Imports is an indication of trade, which is known to dictate the spread of IAS, a reanalysis of the model using Merchandise Imports instead of GDP was still performed to verify the findings. Governance correlated with GDP per capita (*r* = .903, *p* < .001) and Globalisation (*r* = .766, *p* < .001); these two variables were therefore removed.

Response variables in regressions were DAISIE 100 scores. Area, Human Population Density, and Road Density were log‐transformed and centered to zero mean to satisfy regression assumptions. An interaction term (GDP × Governance) was also included in this global model after GDP and Governance were centered to zero mean. Diagnostic plots confirmed that assumptions of linear regression were not violated.

Models for all possible parameter subsets were compared in terms of parsimony and prediction on the basis of Akaike Information Criterion (Akaike, 1973) using the function “dredge” within the package MuMIn (Bartoń, 2014) in R (R Core Team 2014). AICc was used since n/K < 40 (Johnson & Omland, 2004). The difference in the AICc values between the top model and other models was calculated (*∆*_*i*_). Models were ranked in order of increasing *∆*_*i*_. Models with *∆*_*i*_ < 6 were considered the “best” set of models (Symonds & Moussalli, 2011).

For graphical analysis, countries were categorized by GDP according to Ward's minimum variance method in R, which maximized the Euclidean distance between each cluster. This process separated countries into categories based on natural breaks in the data, creating robust, internally consistent categories, and reducing the likelihood of results arising due to poor discretization.

The absence of spatial autocorrelation in our model was determined using the R package *ncf* (Bjornstad, 2013). Moran's I was calculated at 250‐km intervals from the residuals of the global model. Values were between −0.4 and 0.3 for all distances up to 5,000 km, and showed no overall trend, suggesting no significant spatial autocorrelation. Spatial autoregressive models were therefore not used.

## 3 RESULTS

### 3.1 Overall governance score

Model selection showed that no single model was overwhelmingly supported by the data (*∆*_*i *_< 6, Table 1). GDP, Governance, and their interaction appeared in the best model, as well as eight of the 10 models with *∆*_*i*_ < 6 (Table 1). The estimated coefficients of these three terms were positive, and their 95% confidence intervals did not overlap zero in seven of 10 models with *∆*_*i*_ < 6 (Table 1). *R* ^2^ values were consistently high across all top models (Table 1), suggesting parameters within this set explained much of the variance in DAISIE 100 scores. Area, Population Density, and Insularity did not consistently appear in the top models (Table 1).

**Table 1:** Best models (*∆*_*I* _< 6) predicting DAISIE 100 scores in Eurasian countries

Model rank	Intercept	Gov:GDP	Governance	GDP	Area	Insularity	PopDen	Road density	Continentality	Precipitation	Temperature	*K*	Log‐likelihood	AICc	*∆*_*i*_	Adj. *R* ^2^
1	10.67 ± 1.21	4.98 ± 2.83	4.87 ± 4.49	2.94 ± 0.79	NA	NA	NA	NA	NA	NA	NA	4	−101.20	214.3	0	0.667
2	11.11 ± 1.35	4.99 ± 2.79	5.71 ± 4.59	2.72 ± 0.84	NA	+	NA	NA	NA	NA	NA	5	−100.07	214.9	0.59	0.677
3	10.69 ± 1.21	4.43 ± 3.04	4.60 ± 4.52	2.89 ± 0.80	NA	NA	0.60 ± 1.20	NA	NA	NA	NA	5	−100.61	215.9	1.67	0.668
4	11.17 ± 1.35	4.34 ± 2.98	5.48 ± 4.58	2.63 ± 0.85	NA	+	0.70 ± 1.18	NA	NA	NA	NA	6	−99.23	216.2	1.92	0.681
5	10.68 ± 1.22	4.71 ± 2.92	3.80 ± 5.17	3.35 ± 1.24	−0.49 ± 1.15	NA	NA	NA	NA	NA	NA	5	−100.77	216.3	1.99	0.665
6	11.13 ± 1.36	4.70 ± 2.87	4.59 ± 5.21	3.15 ± 1.26	−0.52 ± 1.13	+	NA	NA	NA	NA	NA	6	−99.56	216.9	2.59	0.676
7	11.44 ± 1.40	NA	10.44 ± 4.84	NA	2.78 ± 0.93	+	3.98 ± 1.35	NA	NA	NA	NA	5	−101.75	218.2	3.95	0.647
8	11.27 ± 1.37	3.66 ± 3.42	8.73 ± 9.07	0.95 ± 4.14	1.79 ± 4.30	+	2.51 ± 4.52	NA	NA	NA	NA	7	−98.79	218.5	4.28	0.679
9	10.70 ± 1.23	4.11 ± 3.47	6.10 ± 8.76	2.10 ± 4.02	0.86 ± 4.27	NA	1.46 ± 4.46	NA	NA	NA	NA	6	−100.51	218.8	4.49	0.659
10	10.84 ± 1.29	NA	9.88 ± 4.98	NA	3.07 ± 0.90	NA	4.15 ± 1.49	NA	NA	NA	NA	5	−103.68	219.2	4.97	0.621

Models ranked by increasing AIC_*c*_. Coefficient estimates and 95% CI shown. Gov:GDP, Governance‐GDP interaction; PopDen, Population Density; *K*, Number of fitted parameters (including intercept and residual variance); *∆*_*I*_, Difference between AICc value of the best model and other models; *Adj. R* ^*2*^, Coefficient of determination, adjusted for the number of parameters.

Using hierarchical cluster analysis, four categories of country ordered by increasing wealth as measured by GDP were identified. These categories are as follows: Lower (L; US$11–145bn), Middle (M; US$186–416bn), Upper‐Middle (UM; US$690–793bn), and Upper (U; US$1.8–3.2trn).

**Figure 2:**
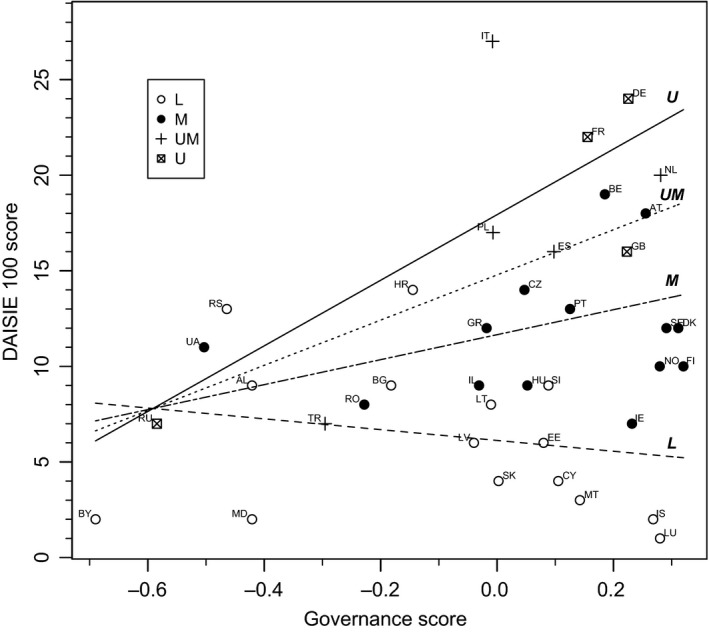
Effect of governance on DAISIE 100 scores was mediated by a country's GDP. Low‐GDP countries (L) showed decreasing scores with increasing governance, as illustrated by the dashed line (‐‐‐) which represents the model output for the lowest GDP country. In contrast, richer countries (U/UM/M) suffered from increasing scores with better governance, as illustrated by the unbroken line (—) which represents the model output for the highest GDP country. Governance scores were centered to mean. Regression lines were drawn from parameter estimates in the best model (*∆*_*I* _= 0, Table 1); M/UM lines used the categories’ median GDP value. Country abbreviations are ISO two‐letter codes: AL (Albania); AT (Austria); BE (Belgium); BG (Bulgaria); BY (Belarus); CY (Cyprus); Czech Republic (CZ); DE (Germany); DK (Denmark); EE (Estonia); ES (Spain); FI (Finland); FR (France); GB (United Kingdom); GR (Greece); HR (Croatia); HU (Hungary); IE (Ireland); IL (Israel); IS (Iceland); IT (Italy); LT (Lithuania); LU (Luxembourg); LV (Latvia); MD (Moldova); MT (Malta); NL (the Netherlands); NO (Norway); PL (Poland); PT (Portugal); RO (Romania); RS (Serbia); RU (Russia); SE (Sweden); SK (Slovakia); SI (Slovenia); TR (Turkey); UA (Ukraine)

These groupings were used to plot Figure 2. The interaction between Governance and GDP was a significant determinant of variation in DAISIE 100 scores (Figure 2), appearing in eight of the 10 best models (*∆*_*i* _< 6, Table 1). Surprisingly, the relationship between DAISIE 100 scores and governance was positive for Eurasian countries with higher GDP, suggesting that, ceteris paribus, increases in governance associated with more severe invasions. Contrastingly, for low‐GDP Eurasian countries (L group nations), better governance might do the opposite, as it associated with reduced DAISIE 100 scores (Figure 2).

### 3.2 Separate governance indicators

In order to examine whether any specific aspect of governance associated with IAS, the analysis was repeated replacing overall governance scores with each of the six indicators (Table 2). Voice and Accountability, Political Stability, Regulatory Quality, and Rule of Law returned similar results to the original regression that used an overall governance score, with the interaction term appearing in the top models (Tables 2 and S2–S5). Government Effectiveness and Control of Corruption also appeared in their most parsimonious models but were not as strong predictors, as they were not included in as many of the top models (Tables S6 and S7). Based on the AIC of the best model, Political Stability and Voice and Accountability were the best predictors of the distribution of IAS, of the six aspects of governance (Table 2).

**Table 2:** Best models (*∆*_I _= 0) predicting DAISIE 100 scores in Eurasian countries for each separate governance indicator

Governance variable	Intercept	GDP	Governance indicator	Gov:GDP	Continentality	Insularity	Area	PopDen	Road density	Temperature	Precipitation	*K*	Log‐likelihood	AICc
Political Stability	11.157	3.368	1.813	1.941	NA	NA	NA	NA	NA	0.248	NA	6	−97.539	209.787
Voice and Accountability	10.665	2.949	2.172	1.994	NA	NA	NA	NA	NA	NA	NA	5	−98.982	209.840
Regulatory Quality	11.344	2.599	1.122	1.655	−0.254	+	NA	NA	NA	NA	NA	7	−99.560	216.853
Rule of Law	10.633	2.874	0.553	1.228	NA	NA	NA	NA	1.187	NA	NA	6	−101.631	217.971
Government Effectiveness	11.666	NA	1.973	NA	−0.364	+	2.772	3.418	NA	NA	NA	7	−100.250	218.233
Control of Corruption	11.268	2.692	0.268	0.950	−0.338	+	NA	NA	NA	NA	NA	7	−100.472	218.678

Coefficient estimates shown. Gov:GDP, Governance‐GDP interaction; PopDen, Population Density; *K*, Number of fitted parameters (including intercept and residual variance).

As a robustness test to confirm the validity of using GDP instead of Merchandise Imports as a proxy for trade, the model was rerun with Merchandise Imports as an explanatory variable. This also showed that Governance and the interaction term appeared in the best model, suggesting that using GDP instead of Merchandise Imports did not affect the findings (Table S8).

## 4 DISCUSSION

Whilst many studies have considered the socioeconomic factors influencing the distribution of IAS (Essl et al., 2011; McGeoch et al., 2010; Pyšek et al., 2010; Westphal et al., 2008), our study explores this relationship between governance and IAS distribution in greater detail. The result of our study clearly shows the importance of countries’ governance as well as GDP in explaining invasive species distributions.

Our finding that stronger governance is associated with the introduction of IAS in Eurasian countries with higher GDP stands in sharp contrast with much of the governance‐biodiversity literature, which typically regards poor governance as a threat to biodiversity (Smith, Muir, et al., 2003; Smith, Obidzinski, et al., 2003). An intuitive explanation as to why our results for Eurasian nations with higher GDP contrast with this body of literature is that governance was a further indirect proxy of propagule pressure—a measure of the number and frequency of individuals released into an area to which they are not indigenous (Lockwood, Cassey, & Blackburn, 2005). Propagule pressure is a key determinant of invasion success, being the most important factor determining exotic bird species richness across Europe (Chiron, Shirley, & Kark, 2009) and exotic avian establishment success across 41 island systems worldwide (Cassey, Blackburn, Duncan, & Gaston, 2005). Governance might increase propagule pressure through its beneficial effects on trade. A country's trade increases when the quality of legal and economic institutions is strengthened (Anderson & Marcouiller, 2002). In turn, increased trade boosts the rate at which propagules are introduced, causing more biological invasions (Westphal et al., 2008). Nevertheless, as trade was included in the model, either directly or through the representation of GDP (see Methods), an effect on the volume of trade cannot alone have resulted with the effect of governance found in our models.

In accordance with our findings, there are many examples of governance facilitating trade to the detriment of controlling IAS. World Trade Organization agreements help tackle corruption and foster stronger governance, thus improving international trade (Aaronson & Abouharb, 2013); but agreements generally lack the mechanisms to internalize the externalities of IAS spread through trade (Perrings, Dehnen‐Schmutz, Touza, & Williamson, 2005). Likewise, EU trade agreements are upheld at the expense of controlling IAS: in 1989, Germany banned the import of live European freshwater crayfish in order to halt the spread of invasive crayfish plague (*Aphanomyces astaci* [Schikora 1906]), but the European Commission successfully argued that this law amounted to a “disguised restriction upon trade” within the EU and was thus not permissible (Commission of the European Communities v Federal Republic of Germany, 1994). In such ways, strong governance and trade can interact to facilitate the spread of IAS within Eurasian countries with higher GDP.

Human movement unrelated to trade also spread IAS. For example, the worldwide airline transportation network has allowed interconnections between geographically disparate but climatically similar regions, facilitating biological invasions (Tatem & Hay, 2007). Although Road Density failed to appear in the set of top models, other studies suggest road networks contribute to the spread of IAS (Vilà & Pujadas, 2001). Poor governance might reduce travel to that country, potentially explaining why better governance associated with increased DAISIE 100 scores in certain countries. Political instability, human rights violations, conflict, and terrorism can harm a nation's tourism industry (Neumayer, 2004). In contrast, Europe's Schengen Treaty—which guarantees free human movement between signatory countries—might be facilitating the spread of IAS (Cobo, Vieira‐Lanero, Rego, & Servia, 2010), but its existence depends upon strong governance in member nations.

Our analysis investigated a type of governance that has been termed “conventional governance” (Ceddia et al., 2014). However, there is a growing appreciation of an alternative form of governance—environmental governance, defined as, “the rules, practices, policies, and institutions that shape how humans interact with the environment” (UNEP 2010). A study modeling the role of governance in determining patterns of land use change under agricultural intensification in six South American countries in 1970–2006 found that these two forms of governance led to alternative outcomes for biodiversity: Higher conventional governance scores led to spatial expansion of agricultural land, whereas higher environmental governance scores (as measured by the Environmental Performance Index [EPI] and the area of protected land) were associated with land‐sparing forms of agricultural intensification (Ceddia et al., 2014). Strong conventional governance did not equate to strong environmental governance, because the former reflects the “conditions necessary for the establishment of operational markets, rather than environmental protection per se” (Ceddia et al., 2014).

The differentiation between conventional and environmental governance offers a novel perspective on previous studies associating high corruption with poor environmental performance (Peh & Drori, 2010; Smith, Muir, et al., 2003; Smith, Obidzinski, et al., 2003; Umemiya et al., 2010) and instead suggests that strong conventional governance can go hand in hand with environmental degradation. Our findings resonate with this standpoint: Strong governance could lead to more IAS through its ability to improve the efficiency with which a nation introduces IAS. Conversely, strong governance does not necessarily mean that existing legislation for tackling IAS has always been effective. For example, the 1951 International Plant Protection Convention has evidently been ineffectual at slowing the spread of pests, and the Convention on Biological Diversity (CBD) requires parties to take actions against IAS, but countries are not sanctioned for failing to comply with these directives (Keller, Geist, Jeschke, & Kühn, 2011). In the case of the EU, while the CBD's Article on IAS was adopted into an EU Strategy for Biodiversity (European Commission 2011), EU legislation targeting IAS only came into effect in January 2015 (Genovesi, Carboneras, Vilà, & Walton, 2014). Furthermore, only 55% of countries that are signatory to the CBD have national legislation relevant to IAS (McGeoch et al., 2010). Even where national IAS policies such as black lists are established, their heterogeneity across countries undermines its effectiveness (García‐de‐Lomas & Vilà, 2015).

Environmental governance was not part of this analysis, as the EPI only began in 2000 (Hsu et al., 2014), which, because of invasion debt (see Methods), is not a sufficiently long period of time to confidently assess the impact of EPI on invasions. However, for the countries under analysis, 2013 EPI scores weakly correlated with 2013 WGI estimates (*r*_s_ = .363, *n* = 38, *p* = .025), further supporting the finding by Ceddia et al. (2014) that strong conventional governance does not equal strong environmental governance. Future analyses would benefit by exploring the environmental dimensions of governance, and we expect that, within GDP groups, countries with better environmental governance will show reduced DAISIE 100 scores.

While the explanations discussed so far address the positive association between governance and DAISIE 100 scores in the majority of Eurasian countries studied (U/UM/M group countries), for countries with the lowest GDP included in the analysis (L group countries), our model indicated a negative relationship, whereby better governance associated with reduced DAISIE 100 scores (Figure 2). We suggest that this relationship might be the result of a balance between GDP (which correlates with trade and thus increases propagule pressure) and governance (which, if effective, might decrease propagule pressure through proper control and regulation of vectors and pathways of introduction). Countries with lower GDP likely have less international trade, which in turn reduces their rate of IAS introduction. This low level of propagule pressure might mean strong governance is capable of stemming the flow of incoming IAS. Strengthening governance may therefore lead to differential outcomes in countries with high or low GDP: in countries with lower GDP, stronger governance might be associated with a reduced environmental footprint, whereas in countries with higher GDP, stronger governance might be associated with greater opportunities to exploit the environment more efficiently. This theory, however, needs to be empirically tested.

Many conservationists assume strengthening governance will assist conservation interventions (Peh, 2013; Smith & Walpole, 2005). Our own results partially support this claim—governance policies in many Eurasian countries of smaller economy appear to have the beneficial effect of reducing the spread of IAS. This hints toward a key gap in current approaches to conservation policy. For example, the recent adoption of EU Regulation 1143/2014 on IAS aims to generate a coordinated response to high‐risk IAS. Such siloed approach is therefore insufficient to attain the highest possible environmental outcomes, and our findings reinforce the call to tackle IAS broadly on multiple fronts, including both socioeconomic, governance, and environmental policy.

A related policy implication is that high‐GDP countries with relatively stronger governance are expected to be more vulnerable to the introduction of new IAS. To overcome this enhanced susceptibility to novel IAS, high‐GDP countries with strong governance should devote greater resources to preventing their introduction.

Our findings suggest that the notion of strong governance as an ally to conservation might mask the true complexity of the relationship between governance and biodiversity loss (Katzner, 2005); favorable conservation outcomes are not always associated with good governance. Whilst current conservation activities have paid attention to drivers of IAS such as trade and human movement, the under‐researched relationship between governance and biodiversity means little action has been taken to address the impact of governance on the spread of IAS. Resolving the interactions between governance and biodiversity will reveal a greater understanding of the socioeconomic drivers of biodiversity loss, allowing future interventions to tackle this underappreciated aspect of conservation.

## CONFLICT OF INTEREST

None declared.

## AUTHOR CONTRIBUTIONS

KS‐HP, PzE, and TA conceived the research. TE constructed the database. TE and TA did the analyses. TE wrote the manuscript with all authors contributing to the discussion.
