# Supplementary material for: Does governance play a role in the distribution of invasive alien species?
Source: Ecol Evol. 2018 Jan 17;8(4):1984–94. doi: 10.1002/ece3.3744 (PMC5817130; doi:10.1002/ece3.3744)
Supplement: Supplementary file 1 [file ECE3-8-1984-s001.docx]

**Table S1**

Pearson’s Correlation matrix for all the explanatory variables. Continent: Continentality; GDP cap: GDP per capita; KOF: KOF Globalisation index; Imports: Merchandise Imports; Pop Den: Human Population Density; Human Pop: Human Population; Road Den: Road Density; Rain: Mean Annual Precipitation; and Temp: Mean Annual Temperature.

|  | Area | | Continent | | GDP | GDP cap | | | KOF | IAS100 | | Imports | | Pop Den | | Human Pop | | Road Den | | Governance | | Rain | Temp | |
| --- | --- | --- | --- | --- | --- | --- | --- | --- | --- | --- | --- | --- | --- | --- | --- | --- | --- | --- | --- | --- | --- | --- | --- | --- |
| Area | 1 |  | |  | | |  |  | | |  | |  | |  | |  | |  | |  |  |  |  |
| Continentality | 0.694 | 1 | |  | | |  |  | | |  | |  | |  | |  | |  | |  |  |  |  |
| GDP | 0.449 | 0.039 | | 1 | | |  |  | | |  | |  | |  | |  | |  | |  |  |  |  |
| GDP capita^-1^ | -0.159 | -0.488 | | 0.183 | | | 1 |  | | |  | |  | |  | |  | |  | |  |  |  |  |
| KOF Globalisation index | -0.259 | -0.496 | | 0.068 | | | 0.681 | 1 | | |  | |  | |  | |  | |  | |  |  |  |  |
| IAS100 | -0.134 | -0.411 | | 0.534 | | | 0.471 | 0.644 | | | 1 | |  | |  | |  | |  | |  |  |  |  |
| Merchandise Imports | 0.112 | -0.236 | | 0.873 | | | 0.374 | 0.308 | | | 0.736 | | 1 | |  | |  | |  | |  |  |  |  |
| Human Population Density | -0.209 | -0.492 | | 0.192 | | | 0.272 | 0.399 | | | 0.507 | | 0.499 | | 1 | |  | |  | |  |  |  |  |
| Human Population | 0.715 | 0.374 | | 0.871 | | | -0.125 | -0.199 | | | 0.253 | | 0.599 | | -0.001 | | 1 | |  | |  |  |  |  |
| Road Density | -0.212 | -0.389 | | 0.145 | | | 0.391 | 0.622 | | | 0.595 | | 0.477 | | 0.699 | | -0.073 | | 1 | |  |  |  |  |
| Governance | -0.370 | -0.587 | | 0.046 | | | 0.873 | 0.844 | | | 0.491 | | 0.292 | | 0.236 | | -0.298 | | 0.434 | | 1 |  |  |  |
| Mean Annual Precipitation | -0.262 | -0.509 | | 0.008 | | | 0.476 | 0.320 | | | 0.256 | | 0.114 | | 0.050 | | -0.204 | | 0.199 | | 0.449 | 1 |  |  |
| Mean Annual Temperature | 0.534 | -0.571 | | -0.119 | | | -0.19 | 0.115 | | | 0.247 | | 0.021 | | 0.473 | | -0.234 | | 0.180 | | -0.076 | -0.051 | 1 |  |

**Table S2**

**Voice and accountability: best models (***∆*_I_ < 2) **predicting DAISIE 100 scores in Eurasian countries**

Models ranked by increasing *AIC_c_.* Coefficient estimates shown. Gov:GDP: Governance-GDP interaction; VA: voice and accountability; Pop Den: Population Density; Precipitation: Mean annual precipitation; Temperature: Mean annual temperature; *K*: Number of fitted parameters (including intercept and residual variance); *∆_I_* : Difference between AICc value of the best model and other models; *w_i_* : Akaike weight.

| Model Rank | Intercept | Gov:GDP | VA | GDP | Area | Insularity | Pop Den | Road Density | Continentality | Precipitation | Temperature | *K* | Log-  Likelihood | AICc | *∆_I_* | *w_i_* |
| --- | --- | --- | --- | --- | --- | --- | --- | --- | --- | --- | --- | --- | --- | --- | --- | --- |
| 1 | 10.66 | 1.99 | 2.17 | 2.95 | NA | NA | NA | NA | NA | NA | NA | 5 | -98.98 | 209.8 | 0.00 | 0.088 |
| 2 | 11.10 | 2.00 | 2.44 | 2.73 | NA | + | NA | NA | NA | NA | NA | 6 | -97.67 | 210.0 | 0.21 | 0.079 |
| 3 | 11.17 | 1.81 | 2.42 | 2.77 | NA | + | NA | NA | NA | NA | 0.16 | 7 | -96.60 | 210.9 | 1.10 | 0.051 |
| 4 | 10.68 | 1.83 | 2.13 | 3.00 | NA | NA | NA | NA | NA | NA | 0.14 | 6 | -98.25 | 211.2 | 1.36 | 0.044 |
| 5 | 11.16 | 1.78 | 2.36 | 2.65 | NA | + | 0.62 | NA | NA | NA | NA | 7 | -96.92 | 211.6 | 1.74 | 0.037 |
| 6 | 10.68 | 1.81 | 2.09 | 2.90 | NA | NA | 0.51 | NA | NA | NA | NA | 6 | -98.50 | 211.7 | 1.87 | 0.034 |
| 7 | 10.68 | 1.85 | 1.78 | 2.94 | NA | NA | NA | 0.61 | NA | NA | NA | 6 | -98.53 | 211.8 | 1.93 | 0.033 |

**Table S3**

**Political stability and absence of violence: best models (***∆*_I_ < 2) **predicting DAISIE 100 scores in Eurasian countries**

Models ranked by increasing *AIC_c_.* Coefficient estimates shown. Gov:GDP: Governance-GDP interaction; PS: Political stability and absence of violence; Pop Den: Population Density; Precipitation: Mean annual precipitation; Temperature: Mean annual temperature; *K*: Number of fitted parameters (including intercept and residual variance); *∆_I_* : Difference between AICc value of the best model and other models; *w_i_* : Akaike weight.

| Model Rank | Intercept | Gov:GDP | PS | GDP | Area | Insularity | Pop Den | Road Density | Continentality | Precipitation | Temperature | *K* | Log-  Likelihood | AICc | *∆_I_* | *w_i_* |
| --- | --- | --- | --- | --- | --- | --- | --- | --- | --- | --- | --- | --- | --- | --- | --- | --- |
| 1 | 11.16 | 1.94 | 1.81 | 3.37 | NA | NA | NA | NA | NA | NA | 0.25 | 6 | -97.54 | 209.8 | 0.00 | 0.092 |
| 2 | 11.16 | 1.98 | 0.80 | 3.16 | NA | NA | NA | 1.13 | NA | NA | NA | 6 | -98.09 | 210.9 | 1.10 | 0.053 |
| 3 | 11.17 | 2.00 | 0.81 | 3.23 | NA | NA | NA | NA | -0.21 | NA | NA | 6 | -98.09 | 210.9 | 1.11 | 0.053 |
| 4 | 11.18 | 2.08 | 0.98 | 3.87 | -0.807 | NA | NA | NA | NA | NA | NA | 6 | -98.34 | 211.4 | 1.60 | 0.041 |
| 5 | 11.60 | 1.84 | 0.79 | 3.01 | NA | + | NA | NA | -0.28 | NA | NA | 7 | -96.86 | 211.4 | 1.66 | 0.040 |
| 6 | 11.43 | 1.86 | 1.98 | 3.24 | NA | + | NA | NA | NA | NA | 0.27 | 7 | -96.96 | 211.7 | 1.87 | 0.036 |
| 7 | 11.19 | 2.16 | 1.41 | 3.25 | NA | NA | NA | NA | NA | NA | NA | 5 | -99.90 | 211.7 | 1.89 | 0.036 |
| 8 | 11.16 | 1.94 | 1.45 | 3.16 | NA | NA | 0.85 | NA | NA | NA | NA | 6 | -98.50 | 211.7 | 1.93 | 0.035 |

**Table S4**

**Regulatory quality: best models (***∆*_I_ < 2) **predicting DAISIE 100 scores in Eurasian countries**

Models ranked by increasing *AIC_c_.* Coefficient estimates shown. Gov:GDP: Governance-GDP interaction; RQ: Regulatory quality; Pop Den: Population Density; Precipitation: Mean annual precipitation; Temperature: Mean annual temperature; *K*: Number of fitted parameters (including intercept and residual variance); *∆_I_* : Difference between AICc value of the best model and other models; *w_i_* : Akaike weight.

| Model Rank | Intercept | Gov:GDP | RQ | GDP | Area | Insularity | Pop Den | Road Density | Continentality | Precipitation | Temperature | *K* | Log-  likelihood | AICc | *∆_I_* | *w_i_* |
| --- | --- | --- | --- | --- | --- | --- | --- | --- | --- | --- | --- | --- | --- | --- | --- | --- |
| 1 | 11.34 | 1.65 | 1.12 | 2.60 | NA | + | NA | NA | -0.25 | NA | NA | 7 | -99.56 | 216.9 | 0.00 | 0.046 |
| 2 | 11.14 | 2.04 | 2.07 | 2.58 | NA | + | NA | NA | NA | NA | NA | 6 | -101.16 | 217.0 | 0.19 | 0.042 |
| 3 | 11.21 | 1.84 | 2.04 | 2.64 | NA | + | NA | NA | NA | NA | 0.20 | 7 | -99.75 | 217.2 | 0.39 | 0.038 |
| 4 | 11.15 | 1.79 | 1.31 | 2.60 | NA | + | NA | 1.09 | NA | NA | NA | 7 | -99.86 | 217.4 | 0.59 | 0.035 |
| 5 | 10.59 | 1.90 | 1.63 | 2.88 | NA | NA | NA | NA | NA | NA | NA | 5 | -102.90 | 217.7 | 0.82 | 0.031 |
| 6 | 10.62 | 1.64 | 0.83 | 2.89 | NA | NA | NA | 1.15 | NA | NA | NA | 6 | -101.55 | 217.8 | 0.95 | 0.029 |
| 7 | 11.20 | 1.78 | 1.92 | 2.51 | NA | + | 0.72 | NA | NA | NA | NA | 7 | -100.30 | 218.3 | 1.48 | 0.022 |
| 8 | 10.61 | 1.71 | 1.57 | 2.96 | NA | NA | NA | NA | NA | NA | 0.18 | 6 | -101.88 | 218.5 | 1.62 | 0.021 |
| 9 | 11.31 | 1.53 | 0.71 | 2.61 | NA | + | NA | 0.83 | -0.21 | NA | NA | 8 | -98.80 | 218.6 | 1.72 | 0.020 |
| 10 | 11.16 | 1.90 | 1.56 | 3.11 | -0.62 | + | NA | NA | NA | NA | NA | 7 | -100.48 | 218.7 | 1.84 | 0.019 |

**Table S5**

**Rule of law: best models (***∆*_I_ < 2) **predicting DAISIE 100 scores in Eurasian countries**

Models ranked by increasing *AIC_c_.* Coefficient estimates shown. Gov:GDP: Governance-GDP interaction; RL: Rule of Law; Pop Den: Population Density; Precipitation: Mean annual precipitation; Temperature: Mean annual temperature; *K*: Number of fitted parameters (including intercept and residual variance); *∆_I_* : Difference between AICc value of the best model and other models; *w_i_* : Akaike weight.

| Model Rank | Intercept | Gov:GDP | RL | GDP | Area | Insularity | Pop Den | Road Density | Continentality | Precipitation | Temperature | *K* | Log-likelihood | AICc | *∆_I_* | *w_i_* |
| --- | --- | --- | --- | --- | --- | --- | --- | --- | --- | --- | --- | --- | --- | --- | --- | --- |
| 1 | 10.63 | 1.23 | 0.55 | 2.87 | NA | NA | NA | 1.19 | NA | NA | NA | 6 | -101.63 | 218.0 | 0.00 | 0.034 |
| 2 | 10.60 | 1.45 | 1.17 | 2.86 | NA | NA | NA | NA | NA | NA | NA | 5 | -103.10 | 218.1 | 0.10 | 0.032 |
| 3 | 11.25 | 1.13 | 0.55 | 2.66 | NA | + | NA | NA | -0.29 | NA | NA | 7 | -100.21 | 218.2 | 0.18 | 0.031 |
| 4 | 10.62 | 1.30 | 1.16 | 2.94 | NA | NA | NA | NA | NA | NA | 0.18 | 6 | -102.01 | 218.7 | 0.76 | 0.023 |
| 5 | 10.63 | 1.23 | 0.42 | 2.95 | NA | NA | NA | NA | -0.21 | NA | NA | 6 | -102.03 | 218.8 | 0.80 | 0.023 |
| 6 | 11.60 | NA | NA | NA | 2.98 | + | 2.06 | 2.08 | -0.50 | NA | NA | 7 | -100.63 | 219.0 | 1.02 | 0.020 |
| 7 | 11.54 | NA | NA | 2.70 | NA | + | NA | NA | -0.44 | NA | NA | 5 | -103.57 | 219.0 | 1.05 | 0.020 |
| 8 | 10.62 | 1.33 | 0.70 | 3.49 | -0.73 | NA | NA | NA | NA | NA | NA | 6 | -102.19 | 219.1 | 1.12 | 0.019 |
| 9 | 10.63 | 1.25 | 1.08 | 2.80 | NA | NA | 0.76 | NA | NA | NA | NA | 6 | -102.21 | 219.1 | 1.16 | 0.019 |
| 10 | 11.01 | 1.44 | 1.47 | 2.64 | NA | + | NA | NA | NA | NA | NA | 6 | -102.22 | 219.2 | 1.19 | 0.019 |
| 11 | 11.04 | 1.22 | 0.85 | 2.66 | NA | + | NA | 1.17 | NA | NA | NA | 7 | -100.73 | 219.2 | 1.23 | 0.018 |
| 12 | 11.49 | NA | NA | 2.68 | NA | + | NA | 1.13 | -0.32 | NA | NA | 6 | -102.25 | 219.2 | 1.23 | 0.018 |
| 13 | 11.11 | 1.27 | 1.51 | 2.69 | NA | + | NA | NA | NA | NA | 0.21 | 7 | -100.75 | 219.2 | 1.27 | 0.018 |
| 14 | 11.69 | NA | 1.79 | NA | 2.79 | + | 3.38 | NA | -0.38 | NA | NA | 7 | -100.78 | 219.3 | 1.33 | 0.017 |
| 15 | 11.23 | 1.03 | 0.26 | 2.67 | NA | + | NA | 0.86 | -0.24 | NA | NA | 8 | -99.39 | 219.8 | 1.78 | 0.014 |
| 16 | 11.11 | 1.22 | 1.40 | 2.54 | NA | + | 0.86 | NA | NA | NA | NA | 7 | -101.03 | 219.8 | 1.82 | 0.014 |
| 17 | 10.65 | 1.10 | 0.09 | 2.94 | NA | NA | NA | 0.99 | -0.16 | NA | NA | 7 | -101.04 | 219.8 | 1.84 | 0.014 |

**Table S6**

**Government effectiveness: best models (***∆*_I_ < 2) **predicting DAISIE 100 scores in Eurasian countries**

Models ranked by increasing *AIC_c_.* Coefficient estimates shown. Gov:GDP: Governance-GDP interaction; GE: Government Effectiveness; Pop Den: Population Density; Precipitation: Mean annual precipitation; Temperature: Mean annual temperature; *K*: Number of fitted parameters (including intercept and residual variance); *∆_I_* : Difference between AICc value of the best model and other models; *w_i_* : Akaike weight.

| Model Rank | Intercept | Gov:GDP | GE | GDP | Area | Insularity | Pop Den | Road Density | Continentality | Precipitation | Temperature | *K* | Log-  likelihood | AICc | *∆_I_* | *w_i_* |
| --- | --- | --- | --- | --- | --- | --- | --- | --- | --- | --- | --- | --- | --- | --- | --- | --- |
| 1 | 11.67 | NA | 1.97 | NA | 2.77 | + | 3.42 | NA | -0.36 | NA | NA | 7 | -100.25 | 218.2 | 0.00 | 0.034 |
| 2 | 11.27 | 1.15 | 0.63 | 2.66 | NA | + | NA | NA | -0.32 | NA | NA | 7 | -100.30 | 218.3 | 0.11 | 0.033 |
| 3 | 11.60 | NA | NA | NA | 2.98 | + | 2.06 | 2.08 | -0.50 | NA | NA | 7 | -100.63 | 219.0 | 0.75 | 0.024 |
| 4 | 11.54 | NA | NA | 2.70 | NA | + | NA | NA | -0.44 | NA | NA | 5 | -103.57 | 219.0 | 0.79 | 0.023 |
| 5 | 11.49 | NA | NA | 2.68 | NA | + | NA | 1.13 | -0.32 | NA | NA | 6 | -102.25 | 219.2 | 0.97 | 0.021 |
| 6 | 10.54 | 1.40 | 1.31 | 2.89 | NA | NA | NA | NA | NA | NA | NA | 5 | -103.87 | 219.6 | 1.38 | 0.017 |
| 7 | 10.59 | 1.15 | 0.71 | 2.90 | NA | NA | NA | 1.17 | NA | NA | NA | 6 | -102.49 | 219.7 | 1.46 | 0.017 |
| 8 | 11.62 | NA | 1.39 | NA | 2.91 | + | 2.69 | 1.30 | -0.38 | NA | NA | 8 | -99.38 | 219.7 | 1.50 | 0.016 |
| 9 | 11.11 | 1.26 | 1.69 | 2.68 | NA | + | NA | NA | NA | NA | 0.23 | 7 | -101.00 | 219.7 | 1.51 | 0.016 |
| 10 | 10.57 | 1.24 | 1.32 | 2.97 | NA | NA | NA | NA | NA | NA | 0.20 | 6 | -102.55 | 219.8 | 1.58 | 0.016 |
| 11 | 11.84 | NA | 3.38 | -2.49 | 5.24 | + | 5.81 | NA | -0.40 | NA | NA | 8 | -99.43 | 219.8 | 1.58 | 0.016 |
| 12 | 10.59 | 1.19 | 0.50 | 2.99 | NA | NA | NA | NA | -0.23 | NA | NA | 6 | -102.58 | 219.9 | 1.64 | 0.015 |
| 13 | 10.84 | NA | NA | 2.92 | NA | NA | NA | 1.86 | NA | NA | NA | 4 | -105.43 | 220.1 | 1.85 | 0.014 |
| 14 | 10.84 | NA | NA | 2.97 | NA | NA | NA | 1.26 | -0.22 | NA | NA | 5 | -104.16 | 220.2 | 1.96 | 0.013 |

**Table S7**

**Control of corruption: best models (***∆*_I_ < 2) **predicting DAISIE 100 scores in Eurasian countries**

Models ranked by increasing *AIC_c_.* Coefficient estimates shown. Gov:GDP: Governance-GDP interaction; CC: Control of Corruption; Pop Den: Population Density; Precipitation: Mean annual precipitation; Temperature: Mean annual temperature; *K*: Number of fitted parameters (including intercept and residual variance); *∆_I_* : Difference between AICc value of the best model and other models; *w_i_* : Akaike weight.

| Model Rank | Intercept | Gov:GDP | CC | GDP | Area | Insularity | Pop Den | Road Density | Continentality | Precipitation | Temperature | *K* | Log-  likelihood | AICc | *∆_I_* | *w_i_* |
| --- | --- | --- | --- | --- | --- | --- | --- | --- | --- | --- | --- | --- | --- | --- | --- | --- |
| 1 | 11.27 | 0.95 | 0.27 | 2.69 | NA | + | NA | NA | -0.34 | NA | NA | 7.00 | -100.47 | 218.7 | 0.00 | 0.033 |
| 2 | 11.60 | NA | NA | NA | 2.98 | + | 2.06 | 2.08 | -0.50 | NA | NA | 7.00 | -100.63 | 219.0 | 0.31 | 0.029 |
| 3 | 11.54 | NA | NA | 2.70 | NA | + | NA | NA | -0.44 | NA | NA | 5.00 | -103.57 | 219.0 | 0.34 | 0.028 |
| 4 | 11.49 | NA | NA | 2.68 | NA | + | NA | 1.13 | -0.32 | NA | NA | 6.00 | -102.25 | 219.2 | 0.52 | 0.026 |
| 5 | 11.72 | NA | 1.57 | NA | 2.74 | + | 3.43 | NA | -0.38 | NA | NA | 7.00 | -100.73 | 219.2 | 0.52 | 0.026 |
| 6 | 10.61 | 1.01 | 0.07 | 3.01 | NA | NA | NA | NA | -0.26 | NA | NA | 6.00 | -102.50 | 219.7 | 1.03 | 0.020 |
| 7 | 11.65 | NA | 1.08 | NA | 2.90 | + | 2.60 | 1.47 | -0.39 | NA | NA | 8.00 | -99.54 | 220.0 | 1.37 | 0.017 |
| 8 | 10.84 | NA | NA | 2.92 | NA | NA | NA | 1.86 | NA | NA | NA | 4.00 | -105.43 | 220.1 | 1.40 | 0.017 |
| 9 | 10.62 | 0.95 | 0.41 | 2.90 | NA | NA | NA | 1.21 | NA | NA | NA | 6.00 | -102.73 | 220.2 | 1.49 | 0.016 |
| 10 | 10.84 | NA | NA | 2.97 | NA | NA | NA | 1.26 | -0.22 | NA | NA | 5.00 | -104.16 | 220.2 | 1.52 | 0.016 |
| 11 | 10.57 | 1.20 | 0.83 | 2.90 | NA | NA | NA | NA | NA | NA | NA | 5.00 | -104.25 | 220.4 | 1.70 | 0.014 |
| 12 | 10.84 | NA | NA | 3.03 | NA | NA | NA | NA | -0.34 | NA | NA | 4.00 | -105.65 | 220.5 | 1.84 | 0.013 |
| 13 | 11.52 | NA | NA | 2.64 | NA | + | 0.69 | NA | -0.37 | NA | NA | 6.00 | -102.93 | 220.6 | 1.89 | 0.013 |
| 14 | 10.60 | 1.04 | 0.90 | 2.98 | NA | NA | NA | NA | NA | NA | 0.20 | 6.00 | -102.98 | 220.7 | 1.99 | 0.012 |

**Table S8**

**Best models (***∆*_I_ < 2) **predicting DAISIE 100 scores in Eurasian countries**

Models ranked by increasing *AIC_c_.* Coefficient estimates shown. Gov:Imports: Governance-Merchanise imports interaction; Imports: Merchanise Imports; PopDen: Population Density; Precipitation: Mean annual precipitation; Temperature: Mean annual temperature; *K*: Number of fitted parameters (including intercept and residual variance); *∆_I_* : Difference between AICc value of the best model and other models; *w_i_* : Akaike weight.

| Model rank | Intercept | Gov:Imports | Governance | Imports | Area | Insularity | PopDen | Road Density | Continentality | Precipitation | Temperature | *K* | Log-Likelihood | AICc | *∆_I_* | *w_i_* |
| --- | --- | --- | --- | --- | --- | --- | --- | --- | --- | --- | --- | --- | --- | --- | --- | --- |
| 1 | 10.40 | 0.25 | 0.15 | 3.13 | NA | NA | NA | NA | NA | NA | 0.24 | 6 | -100.95 | 216.6 | 0.00 | 0.043 |
| 2 | 10.36 | 0.28 | 0.31 | 2.39 | 1.00 | NA | NA | NA | NA | NA | 0.40 | 7 | -99.63 | 217.0 | 0.38 | 0.036 |
| 3 | 11.66 | NA | 0.41 | NA | 2.85 | + | 3.50 | NA | -0.34 | NA | NA | 7 | -99.69 | 217.1 | 0.51 | 0.033 |
| 4 | 10.31 | 0.30 | 0.17 | 3.44 | NA | NA | -1.40 | NA | NA | NA | 0.48 | 7 | -99.77 | 217.3 | 0.67 | 0.031 |
| 5 | 10.42 | 0.24 | -0.02 | 3.11 | NA | NA | NA | NA | -0.24 | NA | NA | 6 | -101.43 | 217.6 | 0.95 | 0.027 |
| 6 | 10.35 | 0.28 | 0.15 | 2.99 | NA | NA | NA | NA | NA | NA | NA | 5 | -102.90 | 217.7 | 1.06 | 0.025 |
| 7 | 10.79 | 0.25 | 0.21 | 2.89 | NA | + | NA | NA | NA | NA | 0.26 | 7 | -100.15 | 218.0 | 1.43 | 0.021 |
| 8 | 10.95 | 0.23 | 0.01 | 2.82 | NA | + | NA | NA | -0.30 | NA | NA | 7 | -100.16 | 218.1 | 1.45 | 0.021 |
| 9 | 10.79 | 0.28 | 0.39 | 2.06 | 1.08 | + | NA | NA | NA | NA | 0.44 | 8 | -98.55 | 218.1 | 1.45 | 0.021 |
